# Retroperitoneal-first dissection approach at the dorsal space for a huge serous cystic neoplasm of the pancreatic tail: a case report

**DOI:** 10.1186/s40792-022-01578-4

**Published:** 2023-01-03

**Authors:** Atsushi Nanashima, Hiroki Takamori, Naoya Imamura, Kousei Tahira, Eiji Kitamura, Masahide Hiyoshi, Takeomi Hamada, Yuuki Tsuchimochi, Hiroyuki Komori, Toshiyuki Kamoto

**Affiliations:** 1 Division of Hepato-Biliary-Pancreas Surgery, Department of Surgery, Faculty of Medicine, University of Miyazaki, 5200 Kihara, Kiyotake, Miyazaki, 889-1692 Japan; 2 Department of Urology, Faculty of Medicine, University of Miyazaki, 5200 Kihara, Kiyotake, Miyazaki, 889-1692 Japan; 3 Department of Surgery, National Hospital Organization Miyakonojo Medical Center, Miyazaki, 885-0014 Japan

**Keywords:** Huge pancreatic neoplasm, Retroperitoneal dissection, Serous cyst adenoma

## Abstract

**Background:**

Large tumors of serous cystic adenomas in the pancreatic body-to-tail severely compress the surrounding organs and retroperitoneal space.

**Case presentation:**

We present a unique surgical challenge for distal pancreatectomy (DP). We present the case of a patient who had a massive mass lesion measuring more than 20 cm in size that had been misdiagnosed as a retroperitoneal tumor by the previous hospital. However, an expert radiologist at our institute diagnosed serous cystadenoma of the pancreas based on imaging characteristics. We decided to perform retroperitoneal space first dissection using a small incision because we were concerned about tumor infiltration or compressive adhesions in important retroperitoneal vessels. We safely attempted distal pancreatectomy by limiting the laparotomy incision step-by-step while securing the main vascular injury of the retroperitoneum. In addition to the ordinary cooperation with urological surgeons, this technique is referred to by the concept of retroperitoneal procedures for minimally invasive surgery in urology.

**Conclusions:**

This approach is useful for lifting resected specimens by prior and wide retroperitoneal dissection, which may lead to safety and the prevention of unexpected vascular injury.

**Supplementary Information:**

The online version contains supplementary material available at 10.1186/s40792-022-01578-4.

## Background

The anterior approach and the operative risks of major vascular injury to the inferior vena cava and renal vessels made dissection of this space or the combined en bloc resection difficult in case of a massive mass lesion located the between hepato-pancreas and the retroperitoneal organs. Securing the surgical margin of the exposed area was also necessary at the bottom of the tumor to avoid tumor recurrence. Kiguchi et al. recently reported the retroperitoneal-first laparoscopic *(RetLap)* approach to preserve the celiac axis prior to distal pancreatectomy with celiac axis resection (DP-CAR) under laparotomy [1]. In this report, the retroperitoneal operative fields seemed to be well and widely recognized, as well as securing major vessels. The dissection or operative procedures of the retroperitoneal cavity are supposed to be an alternative option to reach the dorsal right liver surface, the bottom of the pancreas, the bifurcation of the celiac axis, or the superior mesenteric artery (SMA). In cases of severely invasive or large pancreatic neoplasms, prior dissection of retroperitoneal space is required to secure important vessels or organ injury. Generally, we have co-operated surgery for retroperitoneal organ resections with combined hepato-pancreatic resections, and the value of knowledge sharing has been reported [2]. Urological surgeons can perform scope-assisted retroperitoneal organ resections or dissections by the retroperitoneal approach using minimally invasive procedures at our institutes. We herein report a case of a huge serous cystic neoplasm that originated from the pancreatic tail, severely compressing surrounding organs, including the left-side retroperitoneal organs, which could be successfully resected by applying the prior retroperitoneal space (retroperitoneal approach-first) dissection described in the above concepts.

## Case presentation

A 55-year-old female experienced abdominal distension but had no pain, bowel obstruction, or other clinical symptoms for several years. At the onset of acute gastroenteritis, a large mass lesion was found in the left abdomen on physiological examination and ultrasonography. The patient was referred to our hospital after being diagnosed with a retroperitoneal tumor near the left kidney.

## Diagnostic findings

**Fig. 1:**
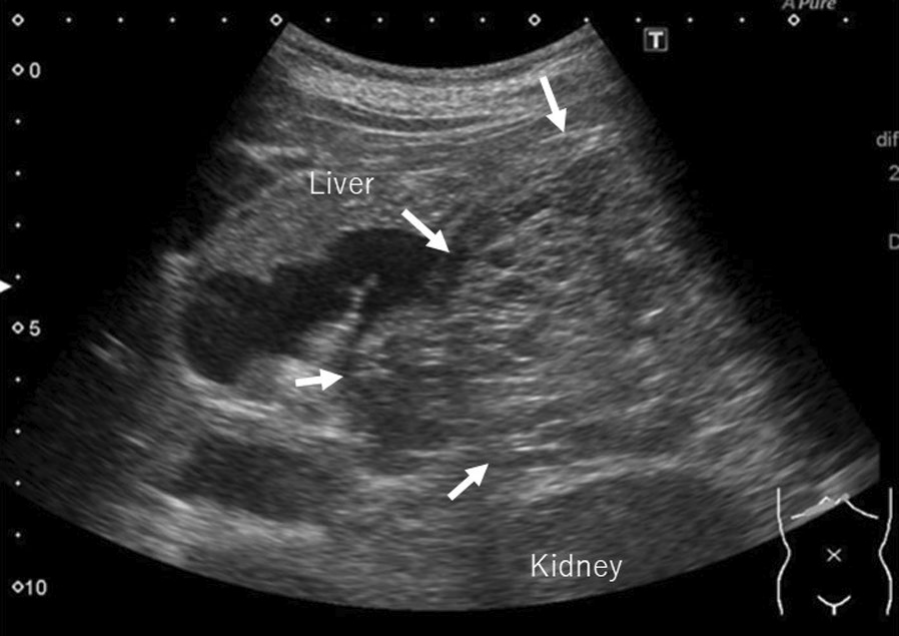
The large irregular mass lesion with a well-defined margin (arrow) compressing surrounding intraabdominal and retroperitoneal organs was seen on extracorporeal ultrasonography. The tumor content showed multi-cystic lesions

**Fig. 2:**
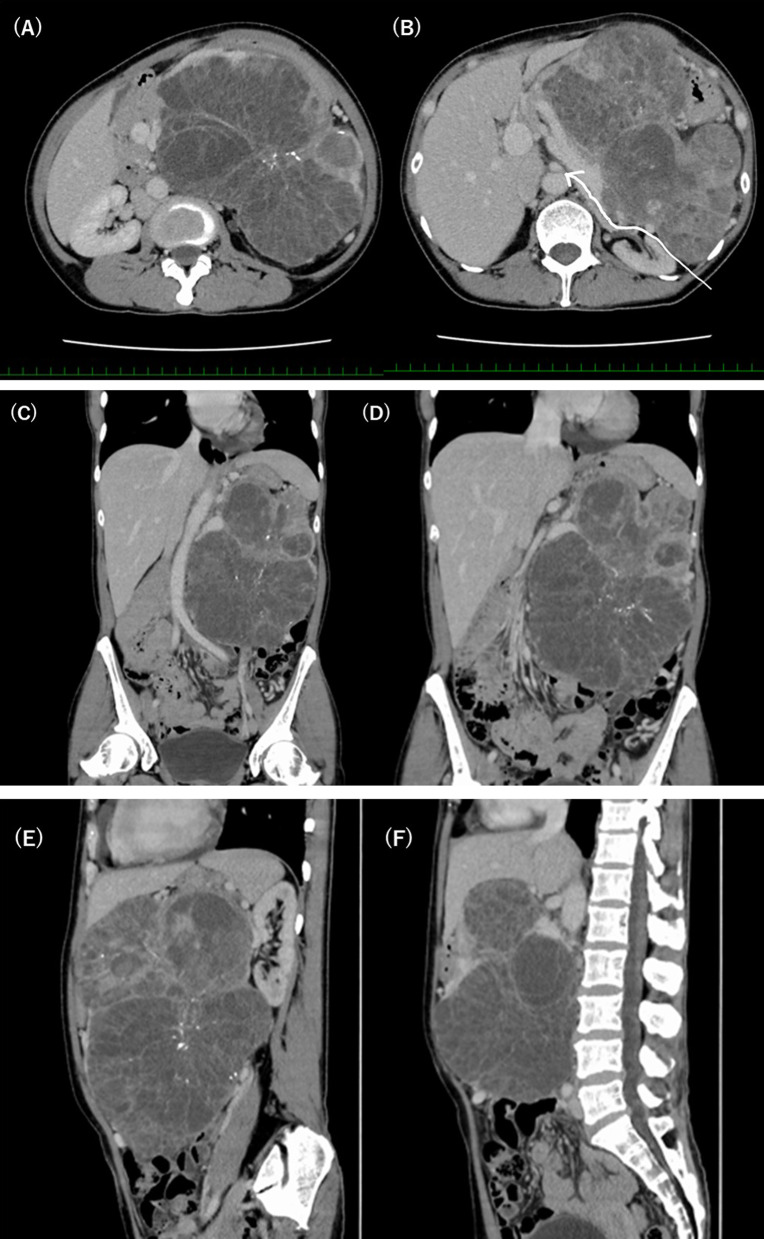
**A** A typical low-density multi-cystic lesion of SCN at the pancreatic tail was revealed by contrast-enhanced CT at porto-venous phase. **B** Tumor dorsally compressed the splenic vein, left renal vessels, and left kidney. The white line indicated the estimated dissected space at the dorsal dissection. **C** Coronary image showed the tumor compression of liver, spleen, abdominal aorta, and left iliac artery. **D** Tumor compressed the superior mesenteric vessels, and intestines. **E** Tumor expanded to both the front of the abdomen and the retroperitoneal space. **F** Tumor spread beyond the level of the abdominal aorta and adjacent vertebra

**Fig. 3:**
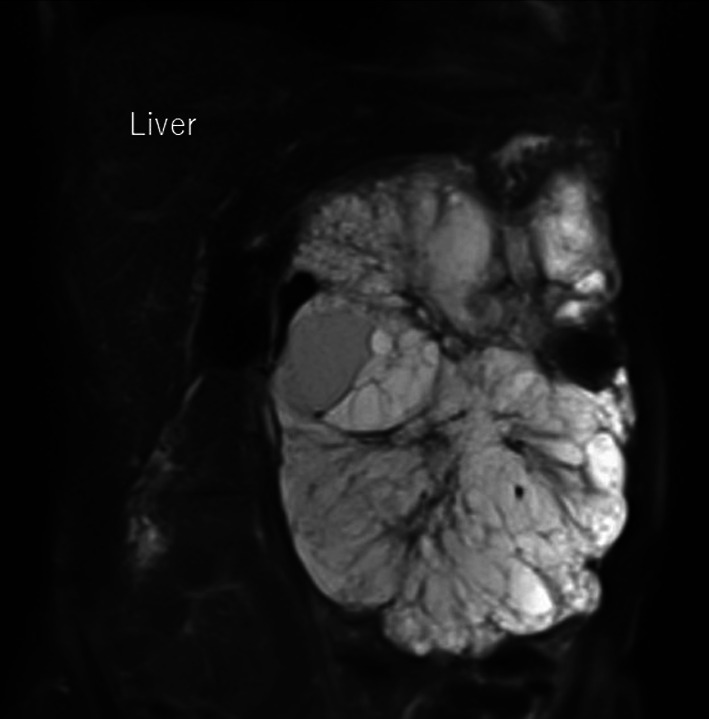
Magnetic resonance T1-intensity image showed the typical high-intensity, multi-cystic tumor lesion of SCN

A tumor exceeding 20 cm in size with central calcification and honeycomb-like heterogeneous content, severely compressing surrounding organs such as the stomach, spleen, left kidney, aorta, vena cava, colon, or small intestines but showing no invasion on ultrasonography (Fig. 1), computed tomography (Fig. 2a–f), and magnetic resonance imaging (Fig. 3), which was radiologically diagnosed as a pancreatic serous cystic neoplasm (SCN). Physical examination revealed the mass was quite palpable. The body was thin, with a height of 153.5 cm and a weight of 47.65 kg (body mass index 20), but there was no cachexia.

## Operative findings

**Fig. 4:**
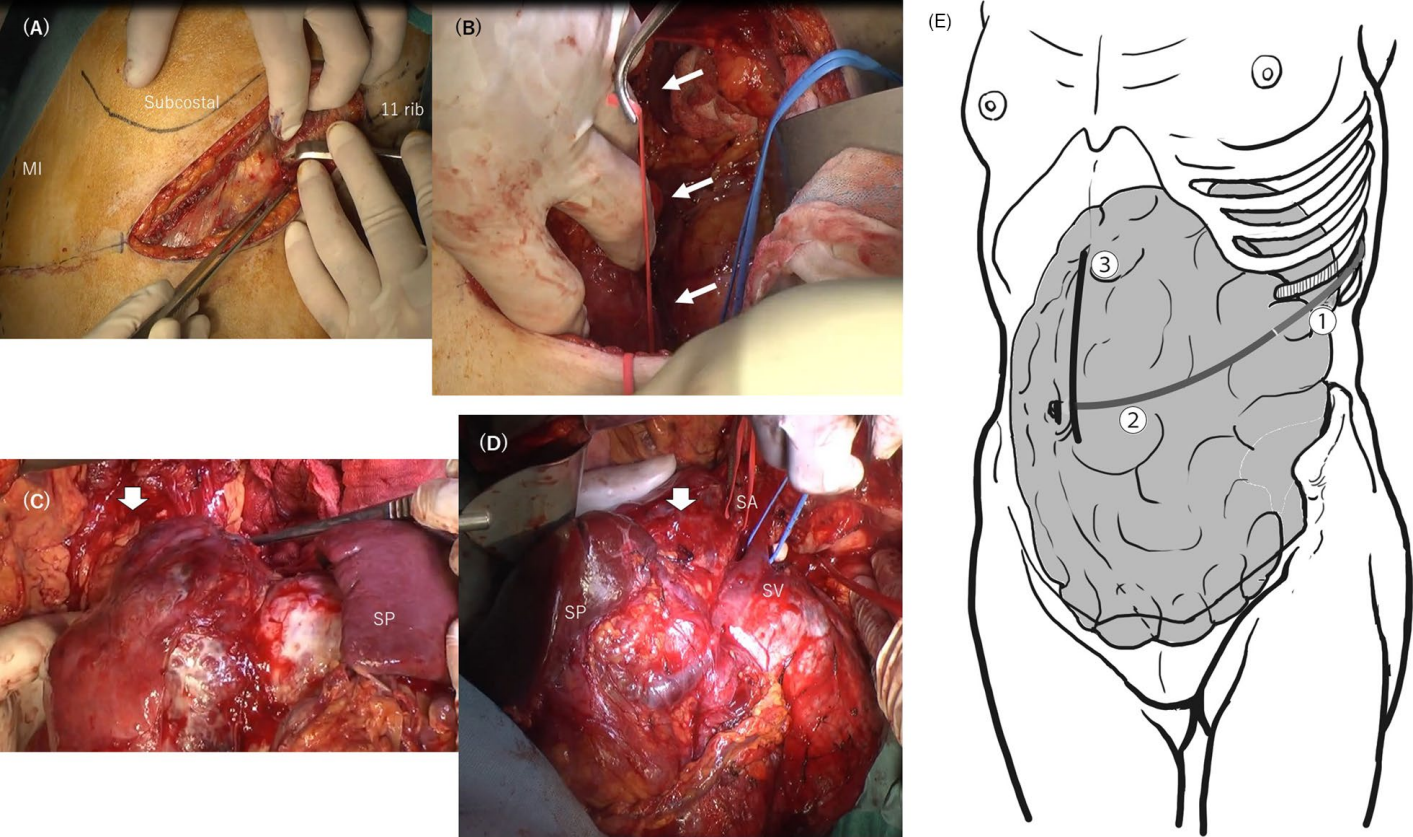
**A** The right lateral decubitus position and the 11th and 12th free ribs were partially resected under the 7 cm in size of incision. **B** The retroperitoneal space was widely dissected near the abdominal aorta (arrows), and the left iliac artery and vein were encircled by vessel tapes (red and blue, respectively). **C** En bloc dissection of the SCN with the spleen (SP) and pancreas body (thick arrow). **D** The tumor could be completely rotated, and the splenic artery (SA) and vein (SV) trunks were taped. The pancreas body was also mobilized. **E** The scheme of step-by-step additional incision by attempting minimum wound. By the right hemi-lateral position, the first retroperitoneal access between the 11th intercostal space was cut as *incision 1* for 7 cm in size of incision. After retroperitoneal dissection, the 12-cm-length *incision 2* up to the vertex of intraabdominal tumor extension was added. To reach the upper and lower tumor edges for removing out of abdominal cavity, the 10-cm-length paramedian *incision 3* was finally required

Based on these findings, the bottom or dorsal tumor margin of the pancreatic fusion fascia was supposed to be a critical bleeding space (the white line in Fig. 2b), and mobilization using only the usual anterior approach under laparotomy was expected to be difficult. Although the *RetLap* minimally invasive procedure was proposed by our group, urological surgeons decided to dissect the retrospective space under the conventional procedure using a 7-cm-long small incision due to the large tumor size (Fig. 4a). The 11th and 12th free ribs were partially resected in the right lateral decubitus position. Urologists confirmed there was no invasion beyond the retroperitoneal fusion fascia after detecting the left kidney, ureter, and renal vessels first. This allowed for wide dissection to the left side of the aorta (Fig. 4b). Subsequently, a lateral incisional laparotomy was performed based on the tumor margin. The tumor extended due to several inflow perfusions. Then, the surrounding adhesive tissues or feeding or drainage vessels from the omental or mesenteric tissues were divided and resected, and eventually the pancreatic body with the tumor and spleen was safely mobilized by lifting from the prior dissecting space (Fig. 4c). The splenic vessels and pancreatic body parenchyma were transected using a linear endostapler (Fig. 4d). The scheme of step-by-step additional incision by attempting minimum wound according to tumor mobilization is illustrated as Fig. 4e. The resected mesocolon was closed, and no organ injury was observed. The operating time was 320 min, and blood loss was 560 mL.

## Histological findings and postoperative course

**Fig. 5:**
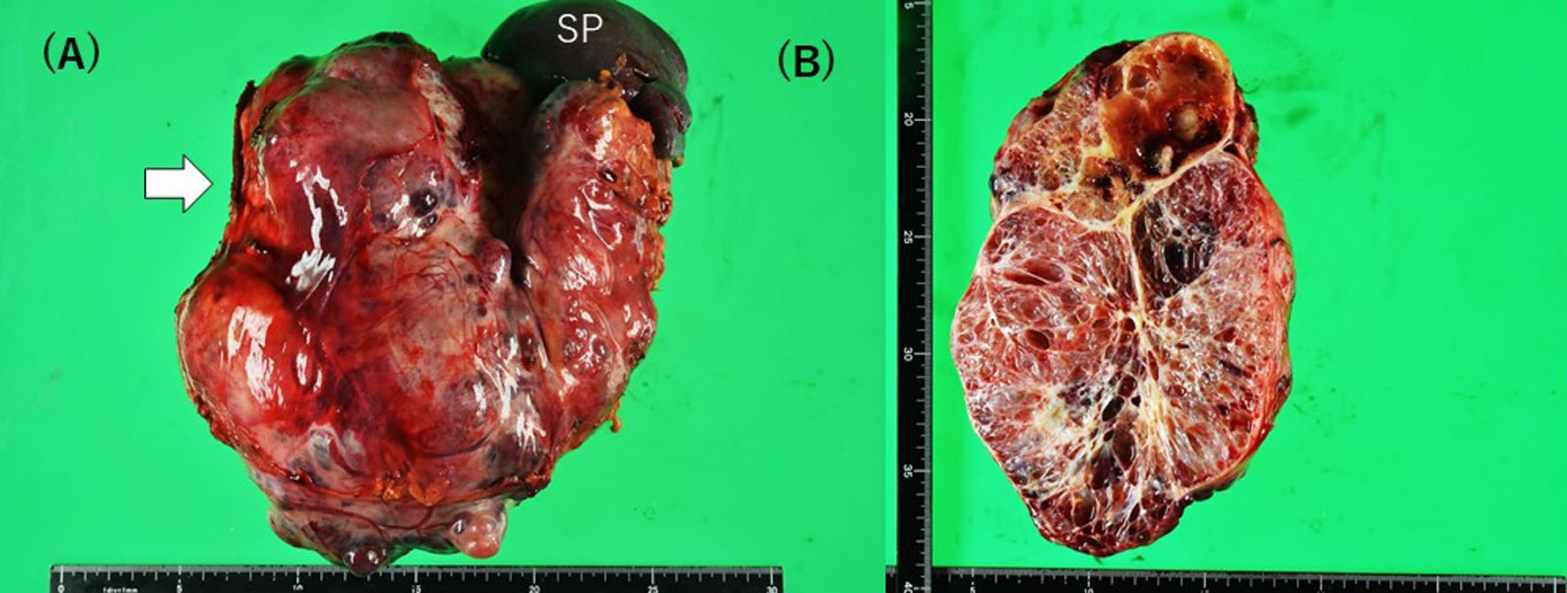
**A** The irregular surface cystic tumor with spleen (SA) and pancreas stump (arrow) in a resected specimen. **B** The transected appearance showed the cysts as cysts and honeycomb-like multi-cysts with hematoma and calcification as partials

Macroscopically, the resected specimen was 20 cm in size and 2.3 kg in mass (Fig. 5a). Multiple cystic lesions were observed with a spongiotic cut surface, and hemorrhagic changes and central scars were partially observed (Fig. 5b). Microscopically, a serous cystadenoma of the microcystic type was diagnosed. The postoperative course was uneventful, without venous thrombosis, and tumor-relapse-free for 14 months at this stage.

## Discussion

The SCN of the pancreas tends to be found as a large mass; however, such a large size with severe compression to surrounding organs seems to be rare [3]. However, the present case showed a mass 20 cm in size with heavy weight in a small body-built female, and such an abdominal space-occupying case was not found in our search using *Pubmed gov* database (https://pubmed.ncbi.nlm.nih.gov/). Interestingly, she had experienced abdominal distension for 3 years; otherwise, no clinical symptoms were observed during this period. In this case, although radical resection could be expected by imaging analysis, a longer operating time and vascular injury to the retroperitoneal or intraabdominal main vasculature were anticipated with the usual anterior laparotomy approach. In cases of large and heavy abdominal tumors, lifting from the dorsal (retroperitoneal) side of the body can make it easier to operate and handle the dissection around the tumor, such as the liver hanging maneuver for a large liver cancer from the front of the vena cava [4], which has also been applied for distal pancreatectomy [5]. Moreover, we often cooperate with urological surgeons for the resection of neoplasms originating from the retroperitoneal organs at our institute [2]. Based on this experience, the prior observation, dissection, or transection of feeding vessels to the tumor via retroperitoneal laparoscopy or incision is very useful for defining the operative indication and subsequent procedures under additional laparotomy. As described above, the *RetLap* procedure seems useful for approaching the para-aortic lesion, and the confluence of the celiac axis, or SMA, can be easily dissected from the surrounding nerve plexus in the field of pancreatic surgery, particularly for DP-CAR under laparotomy [1]. Although the concepts of *RetLap* and urological minimally invasive surgery are different, the combined concepts of this novel procedure for distal pancreatectomy and the conventional urological approach could be widely applied in the field of hepatobiliary–pancreas surgery. In this case, the installation area of the compressive part of the SCN on the retroperitoneal fascia or space was wide, and the tumor was expected to compress the surrounding area chronically for at least 3 years. Vascular injury to this area in a poor operating field is supposed to lead to a lethal situation; therefore, we expected to secure the dorsal space and confirm the possibility of safe dissection using the present approach. In fact, we thoroughly considered the effectiveness of a laparoscopic trial for retroperitoneal dissection in this case, and laparoscopy-assisted dissection was possible in the lateral position in retrospect. If we encounter a similar case, retroperitoneal dissection under laparoscopy would be attempted to find the tumor extension to the retroperitoneal organs and to minimize the length of the incision and its invasiveness for patients. Another advantage of this first retroperitoneal dissection was as follows: we did not care about serious injuries to important retroperitoneal organs or large vessels, and we could lift and mobilize this huge and heavy pancreatic tumor without any hesitation up to pancreatic transection. After this procedure, an additional laparotomy incision was required, except that unexpectedly massive bleeding or organ injury could be avoided, which was safer than the conventional anterior approach (Additional file 1).

## Conclusion

The patient’s postoperative course was uneventful. SCN tends to be found as a large mass, but one such large mass with severely compressed surrounding organs is rare. The retroperitoneal dissection-first approach is less invasive because it secures the dorsal dissection margin and prevents major vascular injury.

## Supplementary Information

**Additional file 1.** The video of operative procedures from the retroperitoneal dissection approach to the distal pancreatectomy for a large SCN of pancreas.

## Data Availability

Not applicable.

## Abbreviations

*RetLap*: Retroperitoneal-first laparoscopic approach
SCN: Serous cystic neoplasm
SMA: Superior mesenteric artery
